# Segmenting Older Adults by Their Acceptance of Digital Health Care Devices: Cross-Sectional Study Using the Augmented Technology Acceptance Model and K-Means Clustering

**DOI:** 10.2196/96557

**Published:** 2026-06-24

**Authors:** Taeyeon Koo, Ill Hyung Jo, Do Young Pyun, Jinho Yoo

**Affiliations:** 1 Office of Research, Chung-Ang University, Seoul, Republic of Korea; 2 Gaedang College of General Education, Sangmyung University, 37, Hongjimun 2-gil, Jongno-gu, Seoul, 03016, Republic of Korea, 82 10 9387 1040; 3 School of Sport, Exercise and Health Sciences, Loughborough University, Loughborough, United Kingdom; 4 Department of Business Administration, Sangmyung University, Seoul, Republic of Korea

**Keywords:** digital health care devices, older adults, technology acceptance model, user segmentation, k-means clustering, principal component analysis

## Abstract

**Background:**

Population aging has become a critical global challenge, with South Korea entering a super-aged society and facing rapidly increasing health care demands. In response, digital health care devices have emerged as promising tools for supporting personalized health management and improving health care accessibility among older adults. However, despite their potential, adoption rates among older adults remain relatively low. Prior research based on the Technology Acceptance Model (TAM) has largely relied on variable-centered approaches, overlooking substantial heterogeneity in acceptance patterns among older adults. A person-centered segmentation approach is therefore needed to identify diverse acceptance profiles. Few studies have integrated the augmented TAM with K-means clustering to identify acceptance-based segments in this population.

**Objective:**

This study aims to segment older adults based on their acceptance patterns toward digital health care devices by integrating the TAM framework with data-driven clustering techniques.

**Methods:**

A cross-sectional survey was conducted with 349 adults aged 65 years and older who were recruited from older adult welfare centers and community facilities in the Seoul metropolitan area of South Korea. We measured 10 constructs within an augmented TAM framework: 2 core constructs (perceived usefulness, perceived ease of use), 6 extended constructs (compatibility, privacy, self-efficacy, price consciousness, health empowerment, attitude toward digital health care), 1 health-related construct (health threat susceptibility), and intention to use as the outcome. Principal component analysis (PCA) and K-means clustering were used to identify latent segments. The number of components was determined using parallel analysis and the Kaiser criterion, and the optimal number of clusters was validated using the silhouette coefficient. Robustness was further assessed through 100-seed stability analysis and PCA sensitivity tests.

**Results:**

We identified 2 principal components, and a 4-cluster solution was selected (K=4, silhouette coeffficient=0.383). The analysis revealed 4 distinct segments: core adopters (57/349, 16.3%), who scored highest across all constructs; potential adopters (64/349, 18.3%), who recognized the value of digital health care devices but exhibited low self-efficacy and perceived ease of use; neutral majority (159/349, 45.6%), who showed near-average scores; and rejecters (69/349, 19.8%), who scored negatively across all dimensions. Robustness checks confirmed high clustering reliability (94%‐99% agreement). Notably, potential adopters represented a critical target group, as their acceptance barriers stemmed from capability constraints rather than lack of motivation. This group combined high perceived usefulness (+0.50) with the lowest self-efficacy (−1.07) and perceived ease of use (−0.83).

**Conclusions:**

This study demonstrated that technology acceptance among older adults is heterogeneous rather than uniform and highlights the importance of segment-specific strategies. By integrating theory-driven acceptance constructs with unsupervised machine learning, the study provides a practical framework for identifying actionable user segments and designing tailored diffusion strategies. These findings offer important implications for policymakers, technology developers, and health care professionals seeking to facilitate inclusive adoption of digital health care technologies in aging societies.

## Introduction

### Background

Population aging has become a defining global challenge, and South Korea stands at its forefront. Having officially entered a super-aged society (with persons aged 65 years and older now exceeding 20% of the total population), the country is projected to become one of the most highly aged nations in the world by 2050 [1]. The old-age dependency ratio, which represents the proportion of older adults relative to the working-age population, is projected to reach 118.5 per 100 working-age individuals by 2072 [2]. These demographic shifts are closely linked to escalating health care expenditures and growing fiscal burdens, underscoring the urgent need for a fundamental transition from treatment-centered care toward preventive and early intervention-oriented health care systems [3].

In this context, digital health care devices, including smartwatches, health monitoring applications, and smart biometric instruments, are emerging as comprehensive tools for personalized health management. These devices enable continuous tracking of physical activity, sleep patterns, heart rate, blood pressure, and blood glucose levels while providing tailored exercise programs and medication reminders [4,5]. From a physical activity and exercise science perspective, such devices hold particular promise for promoting regular exercise and self-managed health behaviors among older adults, who face elevated risks of chronic disease, physical frailty, and sedentary lifestyles [6]. Furthermore, digital health care technologies can improve health care accessibility for older and vulnerable populations facing geographical or physical constraints [5].

Despite the growing availability of these technologies, their adoption among older adults remains comparatively low [4,7]. This well-documented adoption gap has prompted extensive research into the determinants of technology acceptance in this population. The Technology Acceptance Model (TAM), first proposed by Davis [8], has been the most widely used framework for explaining technology acceptance through its core constructs of perceived usefulness (PU) and perceived ease of use (PEOU). Numerous extensions of TAM have incorporated additional factors (eg, compatibility [COMP], privacy [PRIV], self-efficacy [SE], and health empowerment [HE]) to better capture the complex nature of technology acceptance in health care contexts [9-12].

However, TAM-based studies have predominantly examined average effects across entire samples, implicitly treating older adults as a homogeneous group despite substantial heterogeneity in health status, digital literacy, and technology attitudes [6,13]. Consequently, the existing literature provides limited insight into how different subgroups within the older adult population may differ in their acceptance patterns or readiness to accept digital health care technologies.

This limitation carries significant practical implications. For industry stakeholders seeking to commercialize digital health care products for older adults, understanding who is most likely to adopt and why is essential for identifying target customers and designing effective market entry strategies. Rogers’ [14] diffusion of innovations theory suggests that adoption proceeds through different segments, from innovators and early adopters to the late majority and laggards, each requiring differentiated approaches. However, few empirical studies have attempted to identify such adoption segments within older adult populations using theory-grounded data.

To address this gap, this study integrated the augmented TAM framework [11] with a data-driven K-means clustering approach to empirically segment older adults based on their acceptance patterns toward digital health care devices. Unlike conventional regression-based analyses that test predefined hypotheses about population-level effects, clustering techniques group individuals according to multidimensional similarity without prior assumptions, which enables the discovery of latent heterogeneity within the data [15,16]. By applying principal component analysis (PCA) and K-means clustering to theoretically grounded constructs derived from the augmented TAM, this study aimed to classify older adults into different acceptance profiles and to characterize each segment in terms of its cognitive, affective, and behavioral attributes.

This study makes three contributions. First, it provides practical insights for identifying target customer segments when commercializing digital health care products for older adults and bridges the gap between academic research and industry application. Second, it demonstrates that technology acceptance among older adults is not a uniform phenomenon but a heterogeneous, multidimensional process, enriching the theoretical understanding of how diverse subgroups within this population perceive and respond to digital health care technologies. Third, it offers a methodological approach by integrating theory-based causal constructs with unsupervised machine learning, illustrating how data mining techniques can complement and extend established acceptance frameworks.

The aim of this study was to identify and characterize latent acceptance segments of older adults toward digital health care devices and to interpret these segments in ways that can inform tailored intervention design. Accordingly, the following 3 research questions guide the analysis: (1) What latent acceptance segments exist among older adults regarding digital health care devices? (2) How do these segments differ across cognitive, affective, health-related, and behavioral dimensions? and (3) Which segments represent the most actionable targets for tailored intervention strategies?

### The TAM and Its Extensions

The TAM, originally proposed by Davis [8], posits that 2 primary constructs, PU and PEOU, shape individuals’ attitudes and behavioral intentions toward technology use. Since its inception, TAM has become one of the most widely validated and applied theoretical frameworks across diverse domains, including information systems, e-commerce, and health care [17,18]. Its parsimony and explanatory power have made it especially attractive for researchers seeking to identify the key determinants of technology acceptance.

However, as TAM was originally developed to explain technology use in organizational settings, its applicability to contexts involving voluntary, consumer-driven adoption has been questioned. Vijayasarathy [11] noted that TAM’s explanatory scope is limited when applied to situations in which consumers make autonomous decisions about technology use, such as online shopping. To address this limitation, Vijayasarathy [11] integrated multiple theoretical perspectives, including the diffusion of innovations theory [14], the theory of reasoned action [19], the theory of planned behavior [20], and social cognitive theory [21], to propose the augmented TAM. This extended framework retains PU and PEOU as core constructs while incorporating COMP, PRIV, security, normative beliefs, and SE, providing a fuller account of consumer acceptance behavior. The augmented TAM demonstrated strong explanatory power, accounting for approximately 72% of variance in consumer intention in the context of online shopping [11].

In health care contexts, TAM has been similarly extended to capture domain-specific factors. Building on earlier validation studies [10,22], researchers have incorporated constructs such as perceived risk, health empowerment, and facilitating conditions to explain older adults’ acceptance of various health technologies [9,12,23]. These extensions have confirmed the robustness of TAM while demonstrating that health care technology acceptance involves a broader range of cognitive, affective, and contextual factors than originally conceived.

Despite these extensions, TAM’s original PU and PEOU constructs have been critiqued for capturing primarily cognitive-rational aspects of technology perception while underrepresenting emotional, social, and contextual dimensions [24]. This limitation is particularly salient for older adults, whose acceptance is often shaped by affective responses (eg, anxiety, self-doubt), social support structures, and situational constraints. This study therefore adopted the augmented TAM [11] to capture a broader range of cognitive, affective, and contextual factors relevant to this population.

### Older Adults’ Acceptance of Digital Health Care Devices

A growing body of research has examined the factors influencing older adults’ acceptance of digital health care technologies, especially wearable devices. Despite the potential of these technologies to support chronic disease management, physical activity promotion, and independent living, their adoption rates among older adults remain notably low. Javdan et al [25], drawing on US data reported by Farivar et al [26], noted that only 3.3% of adults older than 65 years use wearable devices, compared with 17% among those aged 25 years to 34 years, highlighting a substantial age-related digital divide in high-income Western contexts. Comparable population-level estimates for older adults in South Korea remain scarce, underscoring the need for empirical evidence from this rapidly aging society.

A recent meta-analysis by Yang et al [27] synthesizing 41 studies with 11,574 participants confirmed that PU, PEOU, and social influence are significantly and positively correlated with behavioral intention to use health care technology among older adults (*r*=0.607, 0.525, and 0.551, respectively). However, the meta-analysis also revealed high heterogeneity across studies (*I*²>93%), suggesting that the strength of these relationships varies considerably depending on the type of technology, geographic region, and sample characteristics. This heterogeneity is significant, as it points to the possibility that older adults do not respond to technology in a uniform manner but rather constitute a diverse population with varied acceptance patterns.

Several factors beyond the core TAM constructs have been identified as particularly salient for older adults. Self-efficacy, the confidence in one’s ability to use technology, has been consistently reported as a critical determinant, as many older adults experience anxiety and diminished confidence when interacting with digital devices [13,21]. Compatibility with existing lifestyles and routines is also important, as older adults tend to reject technologies that disrupt their established daily patterns [6,14]. Privacy concerns represent another significant barrier, given that digital health care devices collect sensitive personal health data [4,11]. Health empowerment, reflecting an individual’s perceived capability and motivation to manage their own health, has been shown to positively influence acceptance by strengthening older adults’ sense of agency in health management [28,29].

Qualitative and mixed methods studies have further enriched this understanding. Moore et al [30], in a meta-synthesis of 20 qualitative studies on older adults’ experiences with wearable devices, identified motivation, perceived added value, ease of use, and social support as key themes influencing both initial acceptance and sustained adherence. Javdan et al [25], through a mixed methods investigation of psychological barriers, found that perceived complexity and uncertainty were the most significant obstacles, while perceived disability unexpectedly had a positive effect on attitudes, suggesting that health-related needs can serve as motivators for acceptance. Lee and Coughlin [6] proposed a framework of 10 factors (value, usability, affordability, accessibility, technical support, social support, emotion, independence, experience, and confidence), arguing that technology adoption among older adults is not a purely technical issue but a complex phenomenon shaped by individual, social, technological, and delivery-related considerations.

In the Korean context, Kim and Han [7] conducted a comparative study of baby boomers’ acceptance of telemedicine and wearable health care devices, finding that usefulness, convenience, and cost-saving beliefs were significant predictors. Zin et al [12] similarly used an extended TAM with Korean older adults and reported that PU and PEOU significantly influenced attitudes toward smart health watches. These studies underscore the relevance of TAM-based approaches in the Korean health care context while also pointing to the need for more nuanced analyses that can capture the diversity within the older adult population.

Beyond TAM-based constructs, two complementary theoretical lenses help contextualize older adults’ heterogeneous acceptance patterns. The digital divide framework emphasizes structural disparities in access, skills, and meaningful use of digital technologies across age, income, and education strata, with older adults disproportionately affected [31]. Related to this, eHealth literacy—the ability to seek, understand, appraise, and apply health information from electronic sources [32]—has emerged as a critical determinant of whether older adults can effectively engage with digital health care devices. Recent refinements such as the eHealth Literacy 3.0 model [33] expand this concept to include effectiveness, safety, and privacy dimensions directly relevant to wearable and mobile health technologies.

### Cluster Analysis as a Complement to Theory-Driven Acceptance Models

Although TAM and its extensions have been invaluable for identifying the causal determinants of technology acceptance, these models share a fundamental analytical characteristic: They estimate average effects across entire samples. This approach is appropriate for testing theoretical hypotheses about population-level relationships, but it may obscure meaningful variation among subgroups. Among older adults, who exhibit substantial heterogeneity in health status, cognitive ability, technology experience, and socioeconomic background, relying solely on average-based analyses risks overlooking different acceptance profiles that may require differentiated strategies.

Cluster analysis offers a complementary, data-driven approach to this problem. Unlike regression-based models that test predefined hypotheses, clustering techniques such as K-means group individuals according to multidimensional similarity without prior assumptions about group membership, which enables the discovery of latent patterns within the data [15,16]. K-means clustering is one of the most commonly used unsupervised learning algorithms, valued for its computational efficiency and relative ease of implementation [16].

In health care and health informatics research, K-means clustering has been applied to identify patient subgroups based on disease patterns [34], chronic disease self-management behaviors [35], and digital health engagement levels [36]. Sheng et al [36] applied K-means clustering to analyze the engagement patterns of 60 older adults with multimorbidity using a digital health platform over 12 months. Their analysis identified 3 user groups (typical users, least engaged users, and highly engaged users), with engagement levels significantly associated with health outcomes. This study demonstrated the practical utility of clustering for understanding heterogeneous patterns of technology use among older adults.

When combined with PCA for dimensionality reduction, K-means clustering becomes especially effective for analyzing multivariate survey data. PCA addresses the “curse of dimensionality” that can distort Euclidean distance calculations in high-dimensional spaces while preserving the maximum amount of variance from the original variables [37,38]. This PCA–K-means pipeline has been shown to improve clustering quality and interpretability, especially when applied to correlated variables such as those derived from technology acceptance instruments.

From the perspective of innovation diffusion, the identification of separate acceptance segments resonates with Rogers’ [14] adopter categories. Rogers [14] proposed that, within any social system, individuals can be classified as innovators, early adopters, early majority, late majority, or laggards based on their relative timing of adoption. Each category exhibits different characteristics: Innovators are venturesome and cosmopolite, early adopters serve as opinion leaders, the early majority are deliberate, the late majority are skeptical, and laggards are traditional and resistant to change. Although Rogers’ [14] classification was originally based on temporal patterns of actual adoption, the underlying principle that adoption propensity varies systematically across population segments provides a theoretical rationale for applying segmentation analysis to acceptance data. By empirically identifying acceptance segments through clustering and interpreting them in light of Rogers’ [14] framework, researchers can bridge the gap between theory-driven causal analysis and data-driven pattern discovery.

Despite the theoretical compatibility between clustering and Rogers’ segmentation logic, empirical studies integrating theory-based acceptance constructs with data-driven segmentation remain rare, and most existing work relies on the core TAM only or on general user populations. To our knowledge, no prior study has applied PCA and K-means clustering specifically to an augmented TAM framework for older adults’ digital health care acceptance in a non-Western, super-aged society context such as South Korea. This study addresses this gap by applying PCA and K-means clustering to constructs derived from the augmented TAM [11], thereby identifying different acceptance profiles among older adults and providing practical insights for market segmentation and targeted diffusion strategies.

## Methods

### Study Design

This study used a cross-sectional survey design to examine older adults’ acceptance of digital health care devices in South Korea. Reporting followed the STROBE (Strengthening the Reporting of Observational Studies in Epidemiology) guideline for cross-sectional studies; the completed checklist is provided as Checklist 1.

### Recruitment and Participants

Data were collected between March 31, 2025, and April 15, 2025. Participants were adults aged 65 years and older and were recruited from 6 older adult welfare centers and community facilities located in the Seoul metropolitan area of South Korea. These sites were purposively selected through convenience sampling based on existing institutional partnerships between the researchers’ affiliated university and the centers. Older adults with cognitive impairment or related cognitive difficulties that would prevent meaningful completion of the questionnaire were excluded from participation. Questionnaires were distributed and collected on site by trained undergraduate research assistants—third- and fourth-year students majoring in sport science and health management—who had received prior training on the questionnaire content, administration procedures, and collection protocols and who provided clarification when participants requested additional explanation. The questionnaire included a written description of digital health care devices, defining them as electronic devices that measure, record, and support personal health management. These devices are available in forms such as smartwatches, health monitoring applications, and smart biometric instruments, with functions including tracking of physical activity, heart rate, blood pressure, blood glucose, and sleep, as well as providing tailored exercise programs and medication reminders. Upon completion of the survey, participants received a small token of appreciation (a towel valued at approximately US $10, equivalent to about ₩10,000).

A total of 361 questionnaires were collected. Cases that did not meet the age criterion (65 years and older) were first excluded (n=5). Subsequently, cases in which one or more constructs had all items missing were removed (n=5), as construct scores could not be computed. Finally, cases with severely missing data (more than 3 total missing items across all constructs) were excluded (n=2). This resulted in 349 valid cases for analysis. For the remaining cases with partial item-level missingness (ie, 1 or 2 items missing within a multi-item construct), construct scores were computed using the available item mean, a standard approach for Likert-scale surveys [39].

### Measures

This study examined 10 constructs derived from Vijayasarathy’s [11] augmented TAM supplemented by health-related measures. The constructs, measurement items, and source references are summarized in Table 1.

**Table 1.** Measurement constructs, items, and references.

Construct	Items^a^	Reference
Health threat susceptibility (HTS)^b^	I have difficulty going up and down stairs. I feel tired all the time. I stay awake for most of the time in bed. I have constant pain. I feel that no one is close to me. I feel anxious about my health condition.	Hunt et al [40]
Perceived usefulness (PU)	A digital health care device will be of benefit to me. Using a digital health care device will improve my health. The advantage of a digital health care device will outweigh the disadvantage. Overall, using a digital health care device will be advantageous.	Taylor and Todd [41]
Perceived ease of use (PEOU)	Instructions for using a digital health care device will be easy to follow. It will be easy to learn how to use a digital health care device. It will be easy to operate a digital health care device.	Taylor and Todd [41]
Compatibility (COMP)	Using a digital health care device will fit well with the way I live. Using a digital health care device will fit into my lifestyle.	Taylor and Todd [41]
Privacy (PRIV)	My privacy would be protected when using a digital health care device. A digital health care device can be trusted to safeguard my privacy.	Vijayasarathy [11]
Self-efficacy (SE)	I would feel comfortable using a digital health care device. [removed^c^] If I wanted to, I could easily operate a digital health care device on my own. I would be able to use a digital health care device even if there was no one around to show me how to use it.	Taylor and Todd [41]
Price consciousness (PC)	I am willing to go to extra effort to find lower prices. I will shop at more than one store to take advantage of low prices. The money saved by finding low prices is usually worth the time and effort. The time it takes to find low prices is usually worth the effort.	Lichtenstein et al [42]
Health empowerment (HE)^d^	I am able to make a plan to achieve my health management goals. I can try out different ways of overcoming barriers to my health management goals. I can find ways to feel better when I have health problems. I know positive ways to cope with health-related stress. I know what helps me stay motivated to care for my health. I know enough about myself to make health care choices that are right for me.	Park and Park [28]
Attitude toward digital health care (DHA)	Using a digital health care device is a good idea. Using a digital health care device is a wise idea. I like the idea of using a digital health care device. Using a digital health care device would be pleasant.	Taylor and Todd [41]
Intention to use (IU)	I intend to use a digital health care device soon. I intend to use a digital health care device to monitor my fitness and health conditions. I intend to use a digital health care device frequently in the future.	Taylor and Todd [41]

a All items measured on a 5-point Likert scale (1=strongly disagree; 5=strongly agree).

b HTS items were adapted from the Nottingham Health Profile [40].

c SE item 1 was removed following item-level reliability analysis (see the Methods section for details).

d HE items were adapted from the Diabetes Empowerment Scale-Short Form [29] and reworded for general health management contexts following the example by Park and Park [28].

Health threat susceptibility (HTS; 6 items) was measured to assess participants’ perceived vulnerability to health threats. PU (4 items), PEOU (3 items), COMP (2 items), attitude toward digital health care (DHA; 4 items), and intention to use (IU; 3 items) were adopted from Taylor and Todd [41]. PRIV (2 items) was measured using the scale developed by Vijayasarathy [11]. SE was originally measured with 3 items from Taylor and Todd [41]. However, item-level reliability analysis revealed that SE1 (“I would feel comfortable using a digital health care device”) exhibited a low corrected item-total correlation (*r*=0.244) and that its deletion improved the Cronbach α from 0.643 to 0.778. Given that SE1 captured an affective dimension (comfort) conceptually distinct from the behavioral confidence assessed by SE2 (“I could easily operate a digital health care device on my own”) and SE3 (“I would be able to use a digital health care device even if there was no one around to show me how to use it”) and that the interitem correlation between SE2 and SE3 was high (*r*=0.640) while correlations between SE1 and the other 2 items were low (*r*=0.210 and 0.232), SE1 was removed to enhance measurement validity. The final SE measure comprised 2 items (*α*=0.778). HE (6 items) was measured using the Korean version of the Health Empowerment Scale [28], originally based on the Diabetes Empowerment Scale-Short Form [29]. Price consciousness (PC; 4 items) was measured using the instrument developed by Lichtenstein et al [42].

We excluded 2 constructs from the analysis. Normative belief, measured using a combined format of open-ended referent identification and Likert-scale likelihood ratings, exhibited a high missing rate (11.2%) due to the difficulty older adults experienced in naming specific referent persons. The nonstandard measurement structure and substantial missing data rendered this construct unsuitable for the PCA–K-means pipeline. Security, consisting of a single item, was excluded due to conceptual overlap with PRIV.

All items were measured on a 5-point Likert scale (1=strongly disagree; 5=strongly agree). Following the collaborative and iterative translation method [43], 2 bilingual experts translated all items from English into Korean. During this process, 2 HE items were removed for clarity, and 1 COMP item was excluded due to semantic overlap.

### Data Analysis Procedures

The data analysis proceeded in 3 stages: preprocessing, dimensionality reduction, and clustering. All analyses were performed using Python 3.13 with scikit-learn for PCA and K-means clustering and SciPy for statistical computations.

#### Stage 1: Preprocessing

Construct scores were computed as the mean of constituent items for each participant. All scores were then standardized using *z* scores (mean 0, SD 1) to ensure equal weighting across constructs with different scales and variances.

#### Stage 2: PCA

PCA was applied to reduce the dimensionality of the 10 standardized constructs prior to K-means clustering. Since K-means relies on Euclidean distance, the “curse of dimensionality” in high-dimensional spaces can lead to unstable distance calculations and degraded clustering quality [38]. PCA mitigates this by transforming correlated variables into a smaller set of uncorrelated components while preserving maximal variance [37].

The number of components to retain was determined using parallel analysis [44], which compares observed eigenvalues against the 95th percentile of eigenvalues derived from 1000 random datasets of equal dimension. Components were retained only when observed eigenvalues exceeded this threshold, ensuring that extracted components captured more variance than would be expected by chance. This criterion converged with the Kaiser criterion (eigenvalue≥1.0), both indicating 2 components for retention.

#### Stage 3: K-Means Clustering

K-means clustering was performed on the PCA-transformed data. The optimal number of clusters was determined using 2 complementary methods: the elbow method, which identifies the point of diminishing returns in within-cluster sum of squares (WCSS) reduction [45], and the silhouette coefficient, which evaluates both within-cluster cohesion and between-cluster separation on a scale from –1.0 to 1.0 [46].

#### Supplementary Analyses

To assess the robustness of the results, 3 supplementary analyses were conducted. First, the stability of the cluster solution was evaluated by repeating the K-means algorithm across 100 random initializations. Second, the sensitivity of cluster assignments to the number of retained PCA components was examined by comparing solutions with 2, 3, 4, and 5 components. Third, cluster profiles were characterized by computing the standardized mean scores across all 10 original constructs.

### Ethical Considerations

This study was conducted in accordance with the Declaration of Helsinki. The study protocol was approved by the Institutional Review Board of Sangmyung University (SMUIRB, EX-2025‐004) as a substudy of an umbrella research project on convergent research for older adults. Because older adults are considered a potentially vulnerable population, special care was taken throughout recruitment and data collection. Participation was entirely voluntary. Written informed consent was obtained from each participant prior to data collection, after verbal and written explanation of the study purpose, procedures, potential benefits and risks, and the right to withdraw at any time without penalty. All data were deidentified before analysis.

## Results

### Demographic Characteristics

The demographic characteristics of the participants are summarized in Table 2. The sample was predominantly female (277/349, 79.4%), and approximately one-half of the participants (174/349, 49.9%) were aged 76 years to 85 years. Educational attainment varied widely, with high school completion being the most common level (114/349, 32.7%).

**Table 2.** Demographic characteristics of the study participants (n=349).

Variable	Results, n (%)
Gender	
Male	72 (20.6)
Female	277 (79.4)
Age (years)	
65‐75	145 (41.5)
76‐85	174 (49.9)
≥86	30 (8.6)
Education	
No formal education	10 (2.9)
Elementary school	67 (19.2)
Middle school	86 (24.6)
High school	114 (32.7)
College or above	72 (20.6)

### Descriptive Statistics and Reliability

The internal consistency reliability of all 10 constructs exceeded acceptable thresholds: HTS*, α*=0.777; PU, *α*=0.817; PEOU, *α*=0.701; COMP, *α*=0.839; PRIV, *α*=0.887; SE, *α*=0.778; PC, *α*=0.837; HE, *α*=0.806; DHA, *α*=0.864; and IU, *α*=0.887. Descriptive statistics and reliability coefficients are presented in Table 3.

**Table 3.** Descriptive statistics and internal consistency reliability of constructs.

Construct	Items, n^a^	Results, mean (SD; range)	Cronbach α
HTS^b^	6	2.71 (0.75; 1.00-5.00)	0.777
PU^c^	4	3.93 (0.67; 1.50-5.00)	0.817
PEOU^d^	3	3.33 (0.76; 1.00-5.00)	0.701
COMP^e^	2	3.70 (0.78; 1.00-5.00)	0.839
PRIV^f^	2	3.57 (0.89; 1.00-5.00)	0.887
SE^g^	2	3.26 (0.93; 1.00-5.00)	0.778
PC^h^	4	3.57 (0.81; 1.00-5.00)	0.837
HE^i^	6	3.84 (0.57; 2.00-5.00)	0.806
DHA^j^	4	3.98 (0.62; 1.00-5.00)	0.864
IU^k^	3	3.87 (0.79; 1.00-5.00)	0.887

a All items measured on a 5-point Likert scale (1=strongly disagree; 5=strongly agree).

b HTS: health threat susceptibility.

c PU: perceived usefulness.

d PEOU: perceived ease of use.

e COMP: compatibility.

f PRIV: privacy.

g SE: self-efficacy.

h PC: price consciousness.

i HE: health empowerment.

j DHA: attitude toward digital health care.

k IU: intention to use.

### Principal Component Analysis

**Figure 1.**
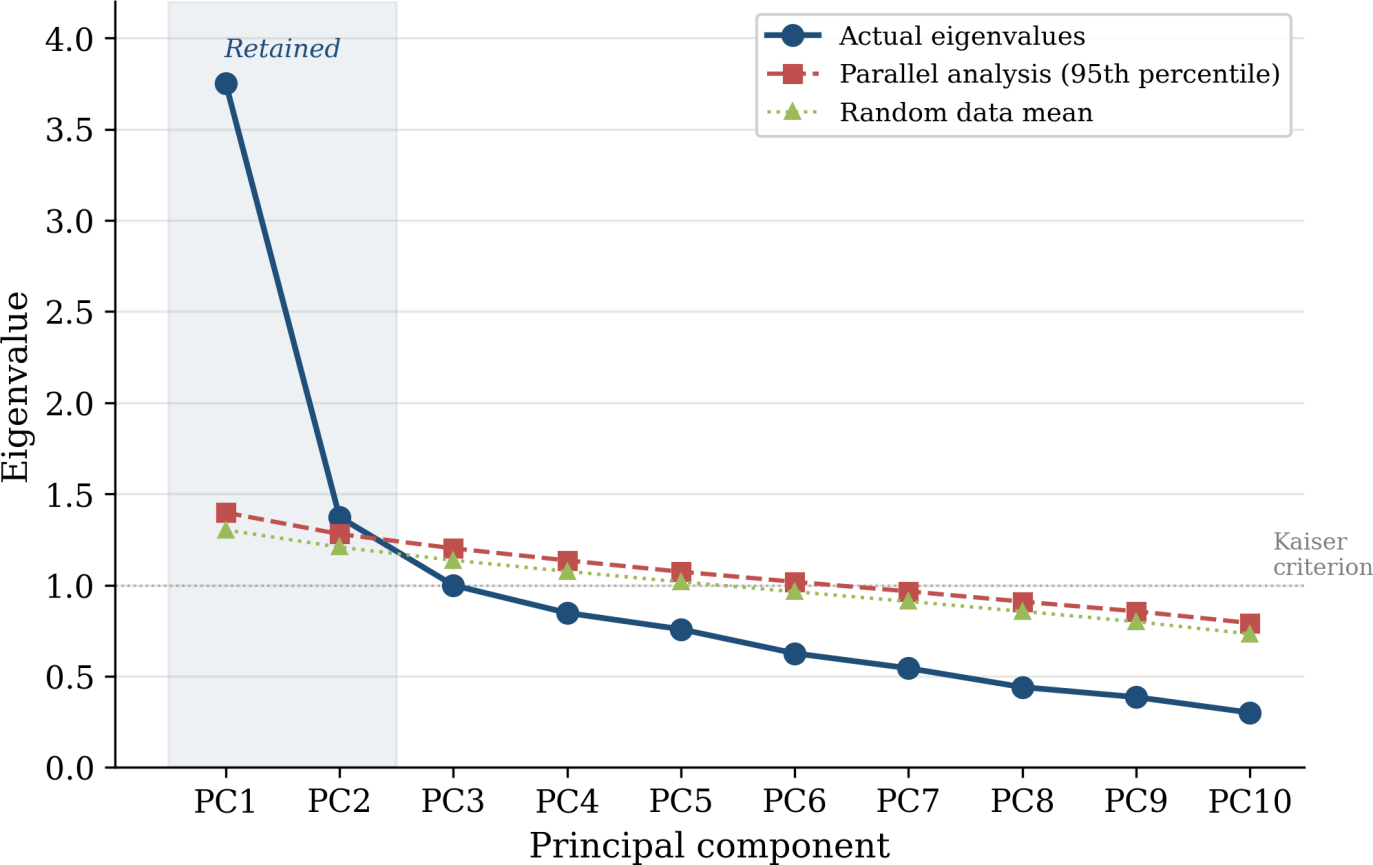
Scree plot with parallel analysis. PC: principal component.

Table 4 presents the eigenvalues from the PCA alongside the results of the parallel analysis. The first component (PC1) had an eigenvalue of 3.751, accounting for 37.40% of the total variance. The second component (PC2) had an eigenvalue of 1.374, explaining an additional 13.70%, yielding a cumulative variance of 51.10%. Beginning with the third component (eigenvalue=0.999), all subsequent eigenvalues fell below the 95th percentile thresholds generated by the parallel analysis (eg, 1.202 for PC3), indicating that these components did not capture more variance than would be expected by chance alone. The Kaiser criterion (eigenvalue ≥1.0) independently confirmed the 2-component solution (Figure 1). Therefore, 2 PCs were retained for subsequent clustering.

**Table 4.** Principal component analysis (PCA) eigenvalues and results of the parallel analysis, which was conducted with 1000 random datasets.

PC^a^	Eigenvalue	Variance, %	Cumulative, %	PA^b^, 95th percentile	Retain^c^
PC1	3.7510	37.40	37.40	1.3979	Yes
PC2	1.3735	13.70	51.10	1.2802	Yes
PC3	0.9993	9.96	61.06	1.2019	No
PC4	0.8479	8.46	69.52	1.1356	No
PC5	0.7582	7.56	77.08	1.0737	No
PC6	0.6262	6.24	83.32	1.0181	No
PC7	0.5445	5.43	88.75	0.9661	No
PC8	0.4409	4.40	93.15	0.9099	No
PC9	0.3861	3.85	97.00	0.8566	No
PC10	0.3011	3.00	100.00	0.7924	No

a PC: principal component.

b PA: parallel analysis.

c Components with eigenvalues exceeding the 95th percentile of random eigenvalues were retained.

The component loadings are presented in Table 5. PC1 showed substantial positive loadings across most acceptance-related constructs (DHA=0.388, IU=0.379, HE=0.365, PU=0.352, and COMP=0.345), representing a general dimension of overall technology acceptance readiness. In contrast, HTS loaded negligibly on PC1 (–0.031), indicating that perceived health threat was largely independent of this general acceptance tendency. PC2 captured a contrast between operational capability and attitudinal motivation: SE (0.573) and PEOU (0.472) loaded positively, whereas HTS (–0.446), DHA (–0.330), and IU (–0.236) loaded negatively. This component thus differentiated individuals who possessed high device operation confidence but relatively lower perceived health needs from those who recognized the value of digital health care but lacked confidence in actual device use.

**Table 5.** Principal component (PC) loadings.

Construct^a^	PC1^b^	PC2^b^
HTS^c^	−0.031	−0.446
PU^d^	0.352	−0.180
PEOU^e^	0.305	0.472
COMP^f^	0.345	0.175
PRIV^g^	0.268	−0.088
SE^h^	0.250	0.573
PC^i^	0.319	−0.080
HE^j^	0.365	−0.088
DHA^k^	0.388	−0.330
IU^l^	0.379	−0.236
Eigenvalue	3.751	1.374
Variance, %	37.40	13.70
Cumulative, %	37.40	51.10

a Two components were retained based on parallel analysis (Horn [44]) and the Kaiser criterion (eigenvalue≥1.0).

b Loadings ≥|0.30| are substantive.

c HTS: health threat susceptibility.

d PU: perceived usefulness.

e PEOU: perceived ease of use.

f COMP: compatibility.

g PRIV: privacy.

h SE: self-efficacy.

i PC: price consciousness.

j HE: health empowerment.

k DHA: attitude toward digital health care.

l IU: intention to use.

### Determination of the Optimal Number of Clusters

**Figure 2.**
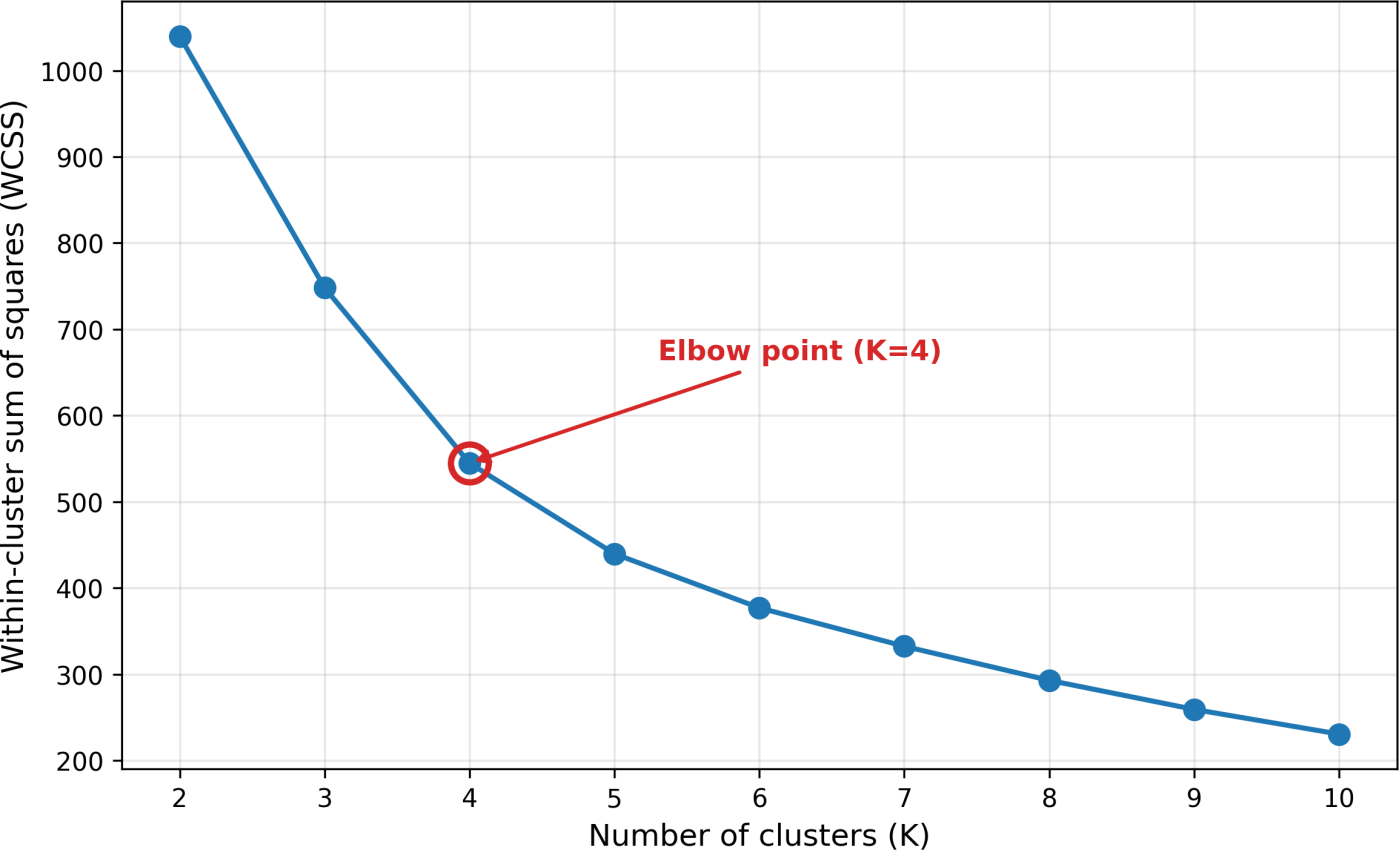
Determination of the optimal number of clusters using the elbow method.

Table 6 presents the WCSS and silhouette coefficients for cluster solutions ranging from K=2 to K=10. The WCSS decreased steadily as K increased; however, the rate of reduction was most pronounced between K=2 and K=4, with drops of 28.06% (K=2 to K=3) and 27.20% (K=3 to K=4), before falling sharply to 19.29% (K=4 to K=5) and <15% thereafter. This pattern indicated a clear inflection point at K=4 (Figure 2).

**Figure 3.**
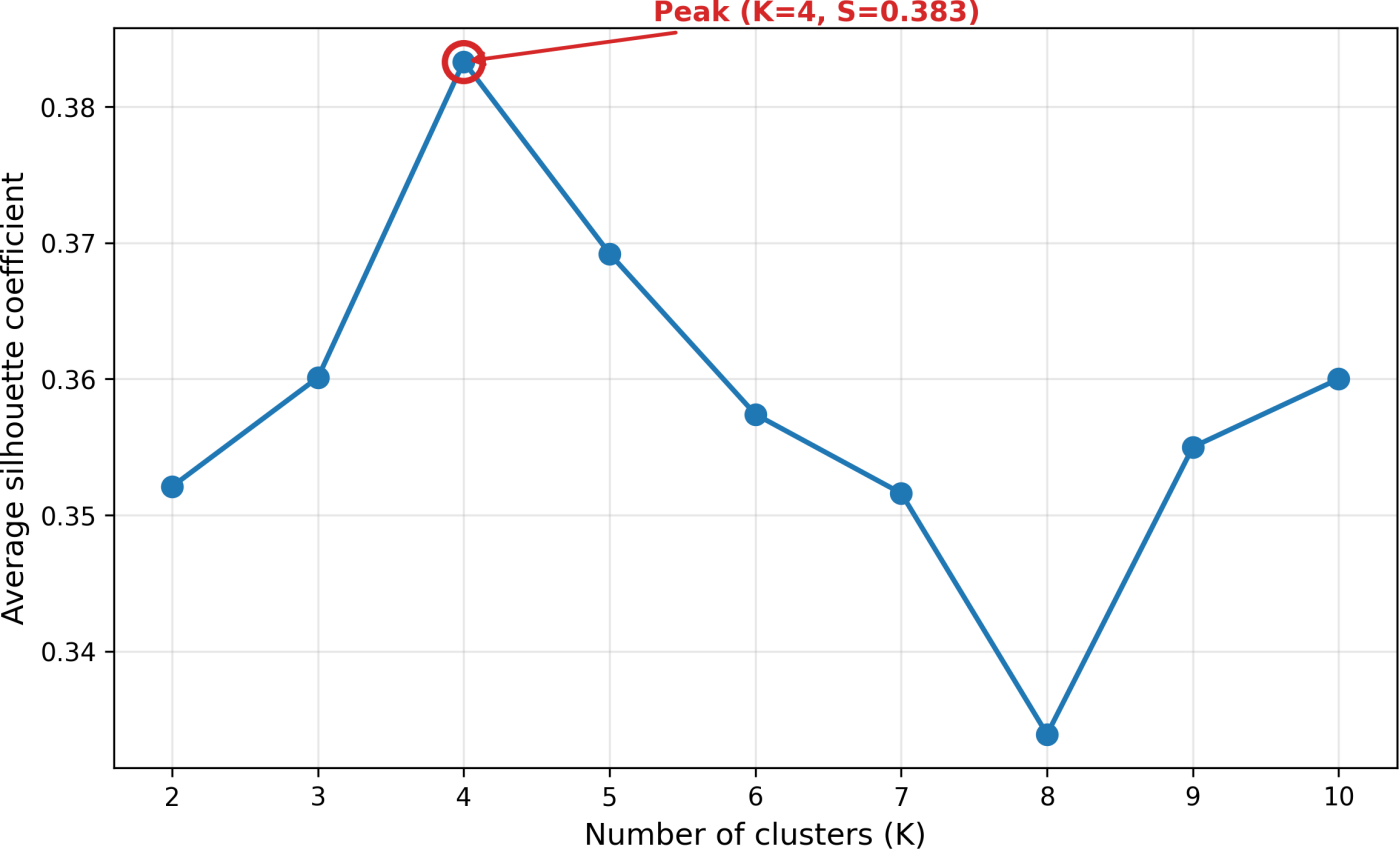
Silhouette (S) coefficients for cluster validation.

The silhouette coefficient reached its maximum at K=4 (silhouette coefficient=0.383; Figure 3), confirming that this solution provided the strongest combination of within-cluster cohesion and between-cluster separation. This convergence of the elbow method and the silhouette criterion supported the adoption of a 4-cluster solution.

**Table 6.** Within-cluster sum of squares (WCSS) and silhouette coefficients for K=2 through K=10.

K	WCSS	ΔWCSS	Drop, %	Silhouette coefficient
2	1039.92	—^a^	—	0.3521
3	748.17	291.76	28.06	0.3601
4^b^	544.68	203.48	27.20	0.3833
5	439.60	105.08	19.29	0.3692
6	376.91	62.69	14.26	0.3574
7	332.33	44.58	11.83	0.3516
8	292.82	39.51	11.89	0.3339
9	259.11	33.71	11.51	0.3550
10	230.49	28.62	11.05	0.3600

a Not applicable.

b Optimal solution. The silhouette coefficient peaked at K=4 (0.383), and the elbow method showed a clear inflection at K=4.

### Cluster Profiles

**Figure 4.**
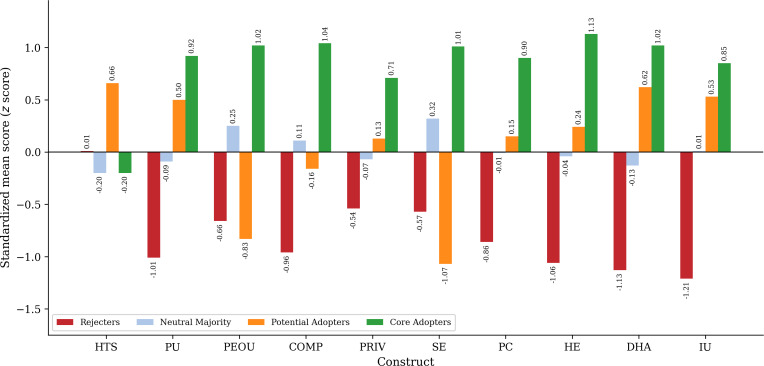
Cluster profiles across 10 acceptance constructs. COMP: compatibility; DHA: attitude toward digital health care; HE: health empowerment; HTS: health threat susceptibility; IU: intention to use; PC: price consciousness; PEOU: perceived ease of use; PRIV: privacy; PU: perceived usefulness; SE: self-efficacy.

**Figure 5.**
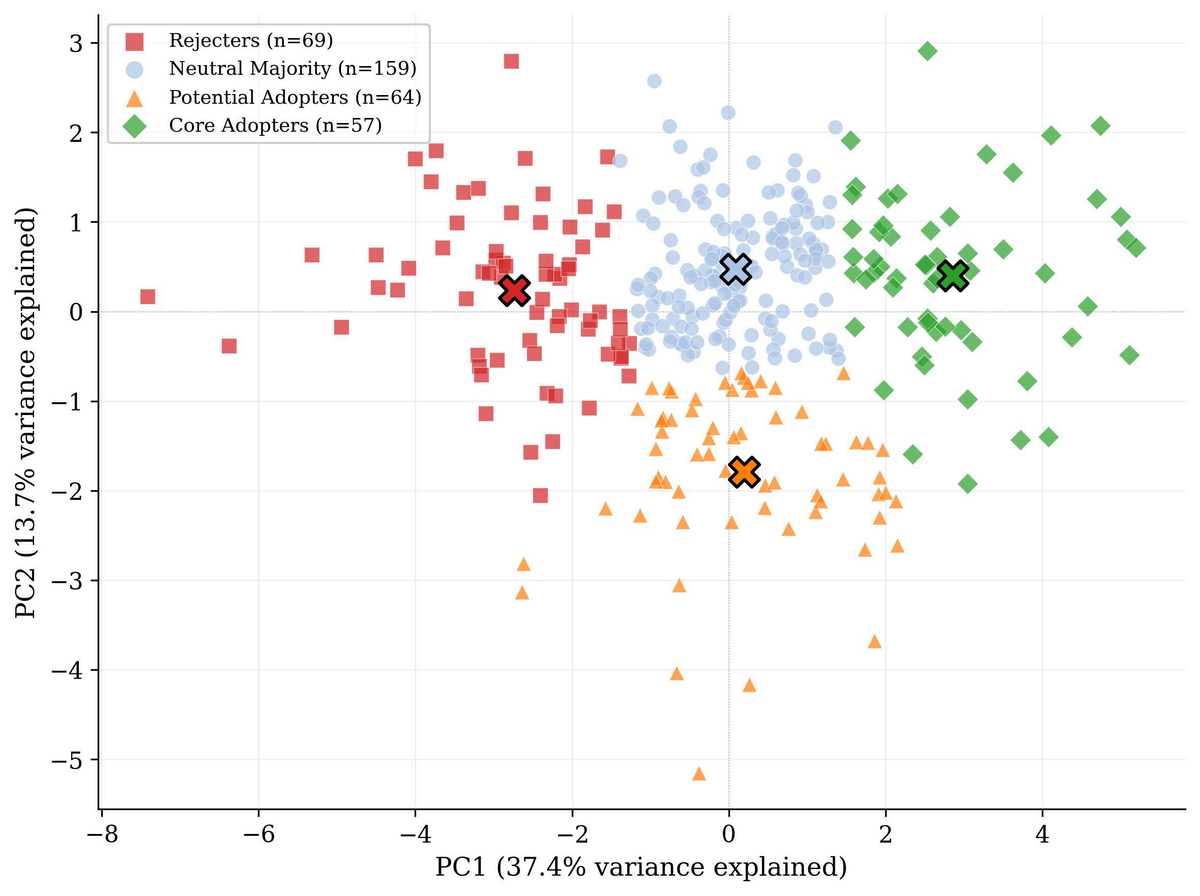
Two-dimensional principal component analysis (PCA) cluster visualization (K=4). PC: principal component.

Figure 4 summarizes the standardized mean scores of each cluster across the 10 constructs. Additionally, Table 7 presents the cluster profiles based on the raw mean scores on a 5-point Likert scale. The 4 clusters were labeled based on their acceptance profiles: core adopters, potential adopters, neutral majority, and rejecters (Figure 5).

**Table 7.** Cluster profiles based on raw mean scores of constituent items (5-point Likert scale: 1=strongly disagree; 5=strongly agree), with segments listed in order of increasing acceptance level.

Construct	Rejecters (69/349, 19.8%), mean	Neutral majority (159/349, 45.6%), mean	Potential adopters (64/349, 18.3%), mean	Core adopters (57/349, 16.3%), mean
Health threat susceptibility (HTS)	2.71	2.56	3.20	2.56
Perceived usefulness (PU)	3.25	3.87	4.26	4.54
Perceived ease of use (PEOU)	2.83	3.52	2.70	4.11
Compatibility (COMP)	2.95	3.78	3.58	4.51
Privacy (PRIV)	3.09	3.51	3.68	4.19
Self-efficacy (SE)	2.73	3.56	2.27	4.19
Price consciousness (PC)	2.87	3.56	3.69	4.30
Health empowerment (HE)	3.24	3.82	3.98	4.49
Attitude toward digital health care (DHA)	3.28	3.90	4.37	4.61
Intention to use (IU)	2.91	3.88	4.30	4.55

**Figure 6.**
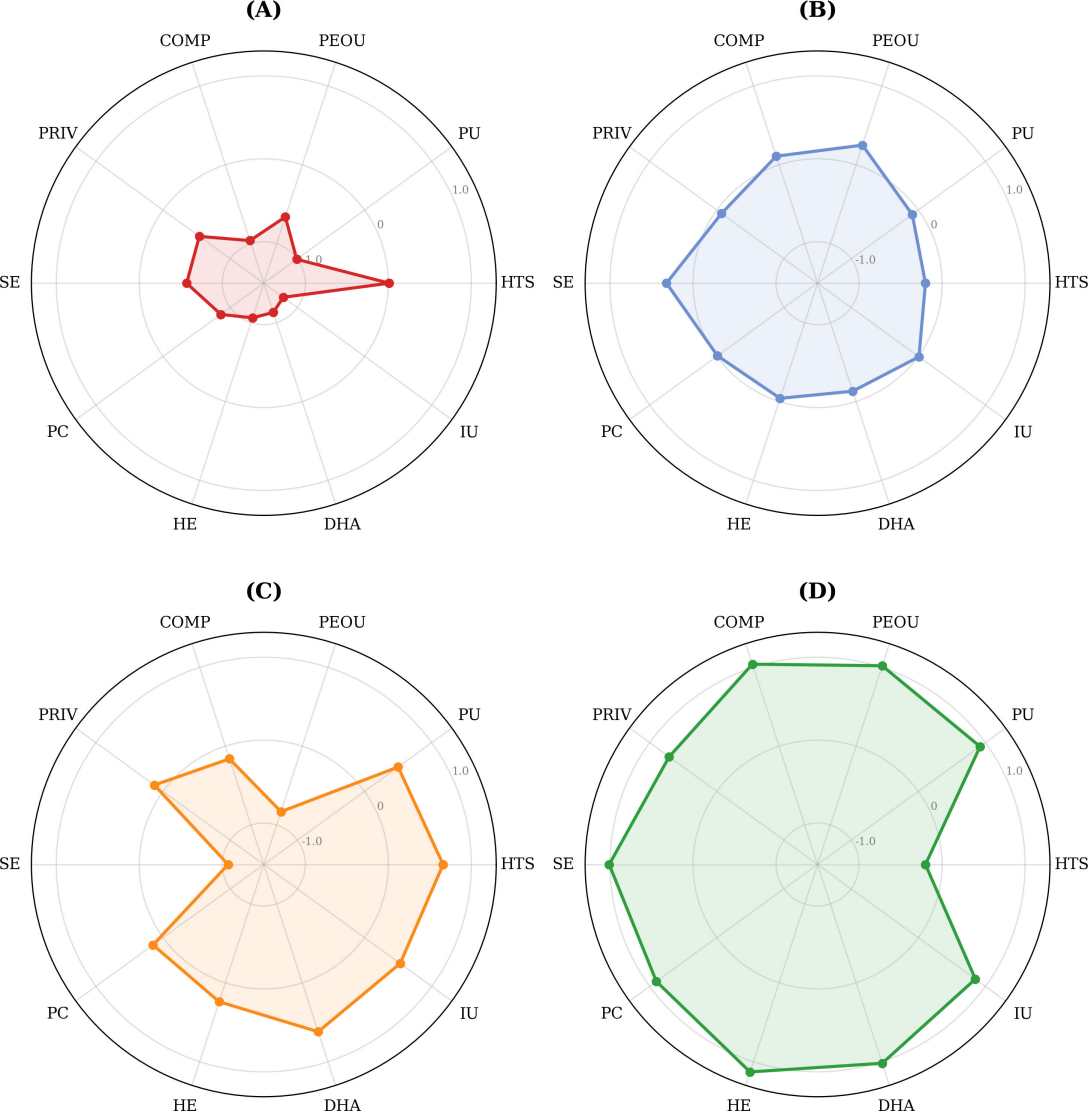
Radar charts of cluster profiles (individual panels; N=349): (A) rejectors (69/349, 19.8%), (B) neutral majority (159/349, 45.6%), (C) potential adopters (64/349, 18.3%), and (D) core adopters (57/349, 16.3%). COMP: compatibility; DHA: attitude toward digital health care; HE: health empowerment; HTS: health threat susceptibility; IU: intention to use; PC: price consciousness; PEOU: perceived ease of use; PRIV: privacy; PU: perceived usefulness; SE: self-efficacy.

Core adopters (57/349, 16.3%) exhibited the highest scores across virtually all constructs (Figures4, 6  7), including PU (0.92), PEOU (1.02), COMP (1.04), SE (1.01), HE (1.13), DHA (1.02), and IU (0.85). Their HTS was below average (–0.20), and their PC was the highest among all clusters (0.90, a pattern consistent with both strong technological readiness and an active health management orientation, possibly reinforced by their lower perceived health vulnerability. This group represented the leading segment most likely to drive initial adoption and serve as opinion leaders in the diffusion process.

Potential adopters (64/349, 18.3%) presented a noteworthy and practically significant profile. They recorded relatively high scores on PU (0.50), DHA (0.62), and IU (0.53), which reflects clear recognition of the value of digital health care devices and a willingness to use these devices. However, their PEOU (–0.83) and SE (–1.07) were substantially below average, the lowest of any cluster. Notably, their HTS was the highest among all clusters (0.66), suggesting that elevated health concerns may serve as a motivational driver for acceptance. This pattern characterizes a “want-to-but-cannot” group: individuals who perceive the need for and benefits of digital health care technologies but lack confidence in their ability to use them.

Neutral majority (159/349, 45.6%) constituted the largest segment, with scores clustering near the overall mean across all constructs. Their PEOU (0.25) and SE (0.32) were slightly above average, while their PU (–0.09), DHA (–0.13), and IU (0.01) were approximately neutral. This group neither strongly embraced nor rejected digital health care technologies, representing a latent majority whose adoption decisions are likely contingent on external factors such as social influence, promotional efforts, or experiential exposure.

Rejecters (69/349, 19.8%) scored negatively across all constructs, with markedly low values for DHA (–1.13), IU (–1.21), HE (–1.06), and PU (–1.01). Their consistently unfavorable profile indicates limited perceived relevance, low confidence, and minimal motivation toward digital health care technologies.

**Figure 7.**
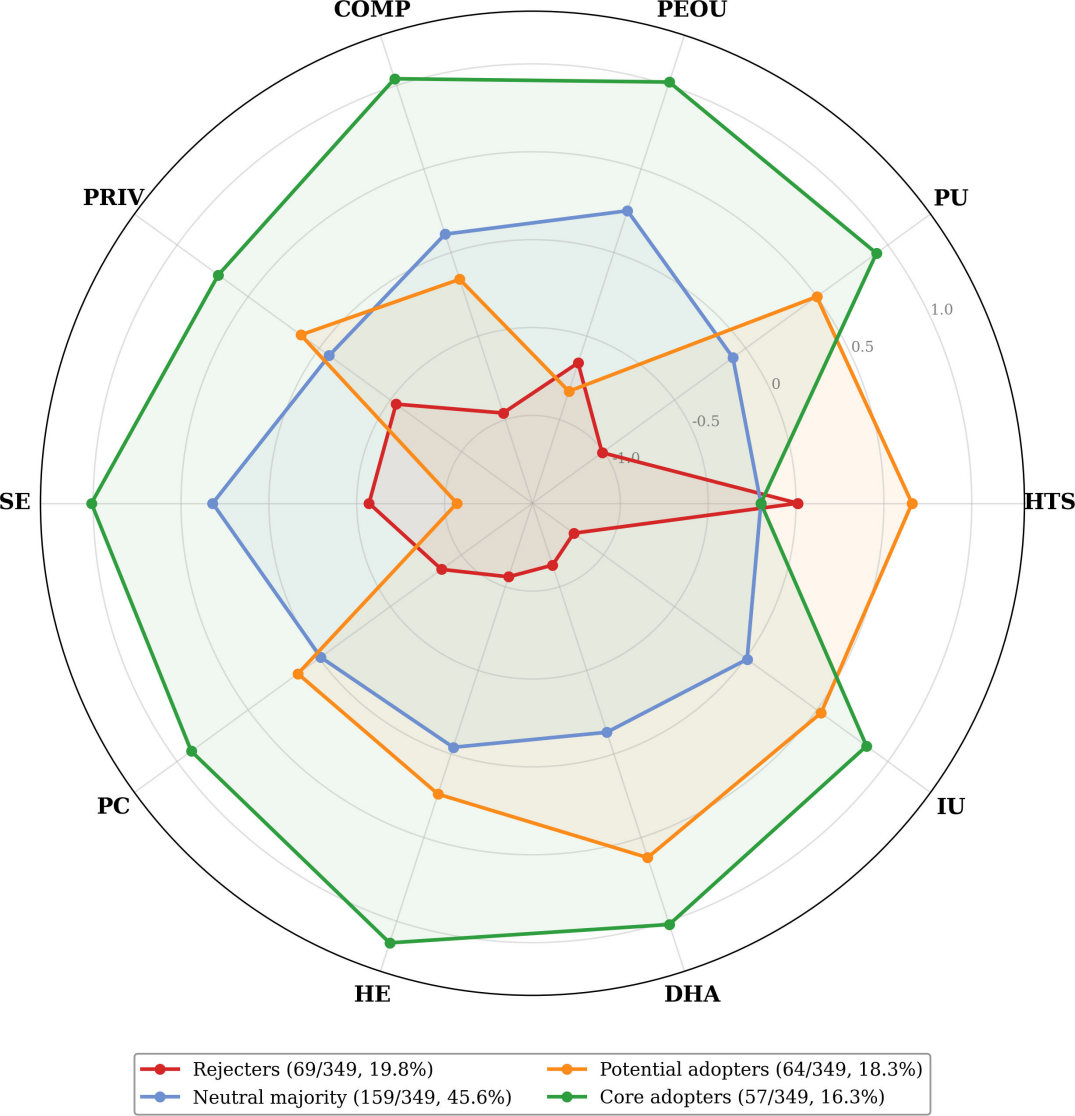
Radar chart of cluster profiles (all segments overlaid; N=349). COMP: compatibility; DHA: attitude toward digital health care; HE: health empowerment; HTS: health threat susceptibility; IU: intention to use; PC: price consciousness; PEOU: perceived ease of use; PRIV: privacy; PU: perceived usefulness; SE: self-efficacy.

### Robustness Checks

**Figure 8.**
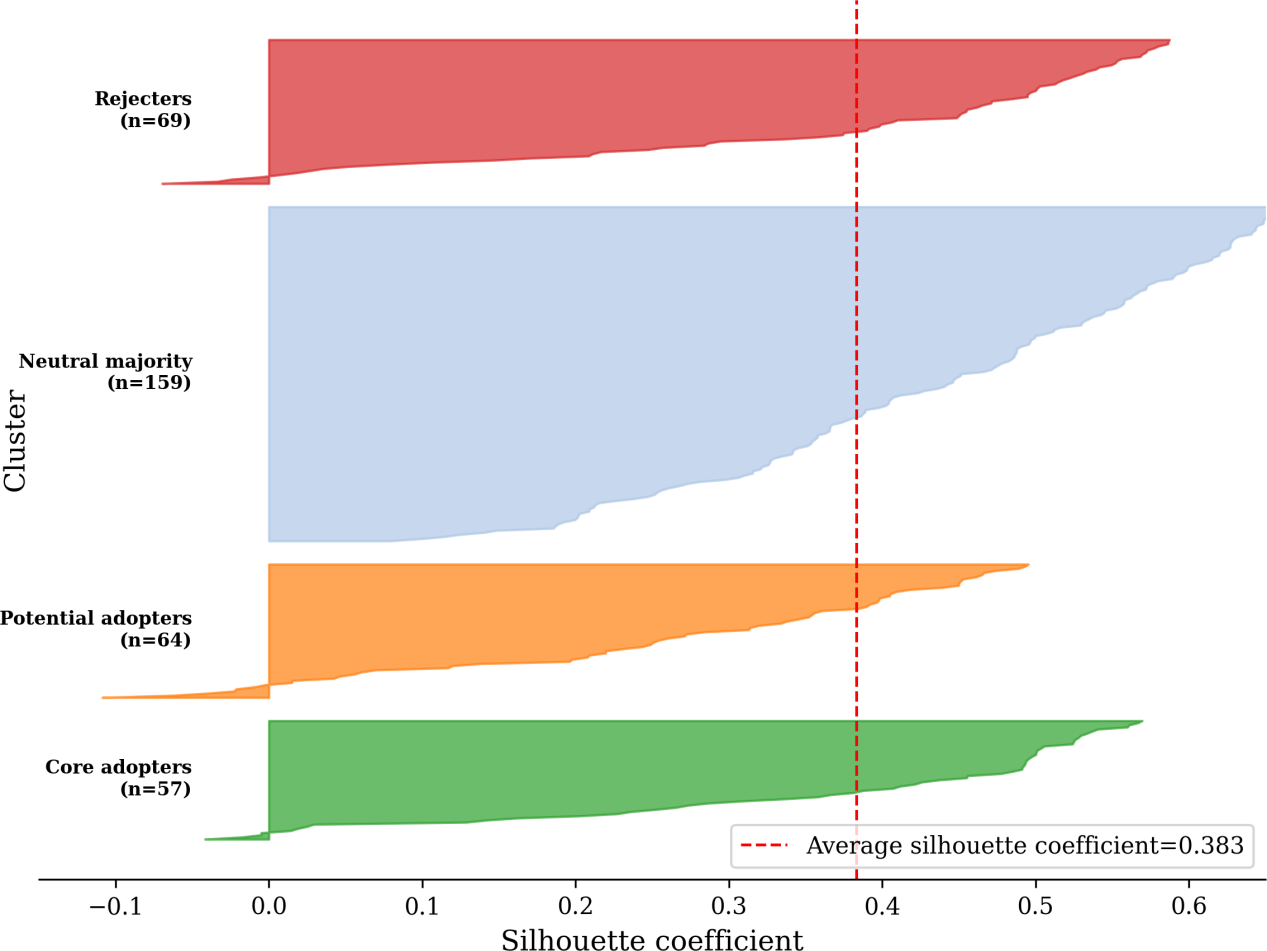
Silhouette plot for the K=4 cluster solution.

Three supplementary analyses confirmed the stability and reliability of the 4-cluster solution. First, the K-means algorithm was repeated across 100 random seed initializations, yielding a mean silhouette coefficient of 0.383 (SD 0.001), indicating high stability with negligible variation across runs (Figure 8).

Second, the sensitivity of cluster assignments to the number of retained PCA components was assessed. When the number of components was increased from 2 to 3, the cluster assignments of 347 of 349 participants (99.4%) remained identical. Increasing to 4 or 5 components still maintained agreement rates of 94.3% (329/349), demonstrating that the identified cluster structure was robust to methodological choices regarding dimensionality reduction.

Third, the cluster solution was examined with respect to demographic characteristics. The distribution of gender, age, and education across the 4 clusters was broadly consistent with the overall sample composition, suggesting that the identified acceptance segments were not merely artifacts of demographic differences but reflected genuine variation in acceptance-related cognitions and attitudes.

## Discussion

### Summary and Interpretation of Findings

This study aimed to segment older adults based on their acceptance patterns toward digital health care devices by integrating the augmented TAM framework [11] with PCA and K-means clustering. The analysis of 349 older adults aged 65 years and older in the Seoul metropolitan area identified 4 acceptance segments: core adopters (16.3%), potential adopters (18.3%), neutral majority (45.6%), and rejecters (19.8%). These results demonstrate that technology acceptance among older adults is not a uniform, linear process but rather a heterogeneous, multidimensional phenomenon in which varied cognitive, affective, and behavioral configurations coexist within the same population.

The PCA revealed 2 interpretable dimensions underlying the 10 acceptance constructs. PC1, accounting for 37.4% of variance, reflected a general technology acceptance readiness factor on which most TAM-derived constructs loaded positively, while HTS loaded negligibly. PC2 (13.7%) captured a contrast between operational capability (PEOU, SE) and attitudinal motivation (DHA, IU, HTS). This 2-dimensional structure suggests that older adults’ acceptance of digital health care devices is shaped by two largely independent forces: (1) an overall evaluative disposition toward technology and (2) a tension between confidence in device use and perceived health-related needs.

The negligible loading of HTS on PC1 is theoretically meaningful rather than incidental. Whereas TAM-derived constructs represent cognitive-evaluative judgments about a technology (its usefulness, ease of use, and compatibility), HTS reflects a self-referential appraisal of one’s own health vulnerability, which is conceptually distinct from any property of the technology itself. Consequently, HTS is unlikely to covary strongly with the general technology-evaluation dimension captured by PC1. Its contribution instead emerged on PC2, where it aligned with motivational-attitudinal constructs (DHA, IU) in opposition to operational capability constructs (PEOU, SE), consistent with its role as a need-based rather than capability-based driver of acceptance.

### Cluster Characteristics in Relation to Theory

The 4 segments identified in this study can be heuristically interpreted through the lens of Rogers’ [14] diffusion of innovations theory, which posits that adoption proceeds through adopter categories (innovators, early adopters, early majority, late majority, and laggards), each characterized by different psychological and social attributes. We note that Rogers’ categories were originally defined by temporal adoption patterns, whereas our segments are derived from acceptance-related perceptions and intentions measured at a single point in time; the correspondence is therefore analogical rather than strict.

The core adopters segment (57/349, 16.3%) closely parallels Rogers’ [14] innovators and early adopters. These individuals scored highest across virtually all constructs, which suggests strong technological readiness, high self-efficacy, and proactive health management orientation. Their elevated PC score (0.90) does not reflect cost aversion but rather an active, informed engagement with the marketplace, consistent with Rogers’ [14] characterization of innovators as individuals who possess substantial resources and the ability to evaluate complex information. Their below-average HTS score (−0.20) suggests that their acceptance is driven not by perceived health vulnerability but by intrinsic technological interest and a proactive approach to health maintenance. From an exercise science perspective, this profile aligns with individuals who are already engaged in self-managed health behaviors such as regular physical activity and who view digital devices as tools to optimize rather than initiate health routines. Beyond self-efficacy, several complementary factors may contribute to this segment’s readiness. Their high HE scores suggest that digital health care devices fit into a pre-existing lifestyle of active health self-management, rather than requiring behavioral change at the point of adoption. Prior experience with consumer technologies may also provide a scaffold that reduces the learning cost of adopting new devices; however, direct measures of such prior experience were not collected in this study.

The potential adopters segment (64/349, 18.3%) represents the most practically significant finding of this study. This group presented a notable contrast: They recognized the value of digital health care devices (PU=0.50, DHA=0.62, IU=0.53) and demonstrated the highest health threat susceptibility among all clusters (HTS=0.66), yet they recorded the lowest SE (−1.07) and PEOU (−0.83) scores. This “want-to-but-cannot” profile suggests that health-related anxiety may function as a motivational driver for acceptance but that confidence barriers prevent this motivation from translating into actual adoption behavior. This finding resonates with the observation by Javdan et al [25] that perceived disability can positively influence attitudes toward wearable devices, suggesting that health needs can paradoxically serve as both motivators and barriers when unaccompanied by sufficient self-efficacy. In Rogers’ [14] framework, this group corresponds to a segment of the early majority whose adoption is contingent on the removal of skill-related barriers rather than persuasion about benefits.

The neutral majority segment (159/349, 45.6%) constituted the largest cluster, with scores clustering near the overall mean across all constructs. Their slightly above-average PEOU (0.25) and SE (0.32) scores, combined with neutral DHA and IU scores, suggested that they possessed basic technological competence but lacked sufficient motivation to actively engage with digital health care devices. This profile is consistent with Rogers’ [14] late majority, who adopt innovations only after the majority of their social system has done so and require strong normative pressure or compelling evidence of benefit. From a market perspective, this group represents the critical mass whose eventual adoption determines whether digital health care technologies achieve mainstream penetration.

The rejecters segment (69/349, 19.8%) exhibited consistently negative scores across all constructs, with markedly low DHA (−1.13) and IU (−1.21) scores. Their low HE score (−1.06) indicated not only resistance to technology but also limited engagement with health self-management more broadly. This profile corresponds to Rogers’ [14] laggards, whose point of reference is the past and whose adoption, if it occurs, typically follows extensive social pressure or institutional mandate. The near-neutral HTS score (0.01) observed in this group suggests that perceived health threats are not particularly salient to them. In contrast to the potential adopters—whose elevated health concerns appeared to drive interest—the rejecters exhibited relative indifference toward health monitoring technologies.

### The Role of HTS

A notable finding of this study, enabled by the inclusion of HTS in the clustering analysis, is the differential role of perceived health vulnerability across segments. The potential adopters’ elevated HTS score (0.66) combined with their high PU and DHA scores but low SE and PEOU scores is consistent with a configural pattern in which health-related concerns co-occur with recognition of potential benefits, while low operational confidence accompanies weaker reported intentions. Because our clustering analysis grouped participants by similarity across constructs rather than testing directional effects, we interpreted this pattern as a possible motivation-capability mismatch rather than a confirmed causal pathway. Longitudinal or experimental designs are required to directly test whether health anxiety drives acceptance and whether low self-efficacy blocks the translation of motivation into use. With this caveat in mind, the pattern nonetheless carries intervention-design implications: For this segment, the more immediate barrier appears to be efficacy-building support rather than further persuasion about device benefits.

In contrast, the core adopters’ low HTS score (−0.20) paired with their high acceptance scores across all constructs indicated that their acceptance was driven by technological self-efficacy and proactive health orientation rather than by perceived health threats. This suggests that, at higher levels of digital competence and active health management, health threat perceptions may become less salient as a driver of acceptance. The substantial heterogeneity (*I*²>93%) reported in the meta-analysis by Yang et al [27] is consistent with this interpretation: If HTS functions as an acceptance driver only when paired with specific configurations of other constructs (eg, low capability, moderate engagement), then sample-wide estimates of HTS-to-intention or PU-to-intention relationships would be expected to vary widely across studies with different sample compositions.

### Practical Implications

The identification of 4 acceptance segments provides practical insights for stakeholders seeking to commercialize digital health care products for older adults or to design public health interventions promoting their adoption. The key practical contribution of this study is that it enables a shift from uniform, one-size-fits-all approaches to targeted, segment-specific strategies.

The potential adopters segment emerged as a particularly promising target for near-term intervention, although this characterization should be understood as an interpretation of the observed configural pattern rather than an empirically tested priority ranking. The rationale for this interpretation is that their motivational profile (high PU score, positive DHA and IU scores) suggests that the resources typically required for attitude change interventions may be unnecessary for this group, so that interventions can concentrate on the capability gap indicated by their low SE and PEOU scores. Practical strategies consistent with this rationale include simplified user interfaces; hands-on training programs conducted in familiar community settings such as older adult centers; and ongoing technical support from trusted individuals including health care professionals, family members, and peer mentors. From a physical activity promotion perspective, integrating digital health care devices into existing community exercise programs (eg, chronic disease management classes or group fitness activities) could simultaneously build device familiarity and reinforce the connection between technology use and tangible health outcomes. Purchase subsidies, rental programs, or insurance-linked incentives could further lower the threshold for initial trial.

The core adopters, although already predisposed to adoption, can serve a catalytic function in the diffusion process. Strategies for this segment should leverage their potential as opinion leaders and early evaluators. Involving them in pilot testing, user feedback initiatives, or peer ambassador programs can generate positive word-of-mouth and create social proof that reduces uncertainty for other segments. Referral-based reward systems and early access programs can further incentivize their role as diffusion accelerators.

The neutral majority segment, as the largest group, represented the critical mass for mainstream adoption. Given their neutral attitudes and moderate competence, strategies should focus on creating experiential exposure opportunities and leveraging social influence. Community-based demonstration events, testimonial campaigns featuring relatable success stories, and integration of digital health care functionalities into public health infrastructure, such as local government health management projects or public-private Smart Healthcare Centers, can gradually shift this group toward active engagement.

The rejecters, given their consistently low acceptance profiles, are unlikely to respond to conventional promotional strategies. A trust-based, long-term approach through gradual exposure via trusted intermediaries (health care providers, family caregivers, or community health workers) is recommended. However, given limited resources, this segment should be treated as a lower-priority target during initial diffusion phases and addressed through long-term inclusion policies in later stages.

### Academic Implications

The academic contributions of this study can be summarized as follows. First, this study deepens the theoretical understanding of older adults’ technology acceptance by demonstrating that this population exhibits heterogeneous acceptance patterns rather than a uniform response to digital health care technologies. The identification of 4 segments, each characterized by unique configurations of cognitive, affective, and behavioral attributes, extends the TAM literature by showing that the constructs identified as significant predictors at the population level combine differently across subgroups. In particular, the discovery of the potential adopters segment, in which high perceived usefulness and positive attitudes coexist with very low self-efficacy and ease of use, challenges the implicit assumption in many TAM studies that positive attitudes naturally lead to intention to use and subsequent behavior. For this subgroup, the pathway from attitude to behavior is blocked not by attitudinal or motivational deficits but by capability gaps, a distinction that is obscured in conventional regression-based TAM analyses.

Second, this study advances methodological approaches by integrating data mining techniques with an established theoretical framework. Although prior TAM research has predominantly relied on structural equation modeling or regression analysis to test predefined causal hypotheses, this study demonstrates how unsupervised machine learning can complement these approaches by revealing latent heterogeneity that hypothesis-driven methods may overlook. The use of parallel analysis [44] for component retention (rather than the commonly used but less rigorous cumulative variance threshold) and the robustness checks (100-seed stability, PCA sensitivity analysis with 94%‐99% agreement rates) represent methodological enhancements that strengthen the credibility of the clustering results. Notably, the finding that the 2-component PCA solution yielded a higher silhouette coefficient (0.383) than solutions with more components challenges the convention of retaining components solely based on variance explained, suggesting that, in clustering contexts, parsimony may be more important than comprehensiveness in dimensionality reduction.

Third, this study bridges the gap between theory-driven acceptance research and practice-oriented market segmentation. Although previous TAM-based studies have primarily identified determinants of acceptance at the aggregate level, this study delineates practical consumer segments that can inform product development, service delivery, and policy intervention. By interpreting the empirically derived clusters through the lens of Rogers’ [14] adopter categories, the study provides a conceptual link between technology acceptance theory and innovation diffusion strategy, offering a scholarly basis for differentiated approaches to digital health care dissemination.

A natural question raised by the radar-chart profiles is whether this 2-step clustering approach yielded insights that a conventional regression-based analysis would not have revealed. Regression models estimate the average effect of each predictor across the entire sample and thus assume that relationships among constructs (eg, between PU and IU) are broadly homogeneous. A regression-based analysis of our data would likely recover the well-established positive associations among TAM constructs but would obscure the configural mismatch observed within the potential adopters segment, in which high perceived usefulness and positive attitudes coexist with very low self-efficacy and ease of use. Because individuals in this subgroup contribute to the sample-wide slope estimates in the same way as more homogeneous profiles, their configural pattern is invisible to variable-centered analyses; clustering makes this heterogeneity explicit by grouping individuals according to multidimensional similarity rather than estimating marginal effects.

### Limitations and Directions for Future Research

Despite its contributions, this study had several limitations that suggest directions for future research. First, participants were recruited through convenience sampling from older adult welfare centers and community facilities in the Seoul metropolitan area. This sampling strategy may overrepresent community-dwelling, socially active older adults and underrepresent homebound individuals, rural residents, or those with cognitive impairment (who were explicitly excluded from the study). In addition, the sample was drawn exclusively from the Seoul metropolitan area and was skewed toward female participants (79.4%), reflecting typical attendance patterns at older adult welfare centers in urban Korea; generalization to male older adults, rural older adults, or older adults in other cultural contexts should therefore be made with caution. Future research should use stratified or probability sampling methods and include participants from diverse geographic and cultural contexts.

Second, this study adopted a cross-sectional design, capturing acceptance patterns at a single point in time. Consequently, it cannot account for temporal changes in attitudes, the intention-behavior gap, or the dynamic process by which individuals may transition between segments over time. Longitudinal or experimental designs would enable researchers to examine how acceptance profiles evolve in response to experiential learning, social influence, or intervention programs.

Third, this study focused on acceptance patterns derived from TAM constructs and did not incorporate broader contextual determinants such as income, health conditions, prior technology experience, family support structures, or health care accessibility. Although the demographic analysis suggested that the identified segments were not mere artifacts of demographic differences, integrating these variables as covariates or moderators could yield richer segmentation profiles and more targeted practical recommendations.

Fourth, the study measured acceptance intentions rather than actual usage behavior. Although behavioral intention is a well-established proxy for adoption in the TAM literature, the relationship between intention and actual behavior is not always straightforward, especially for older adults who may face unanticipated physical, cognitive, or environmental barriers upon actual device use. Future research incorporating objective usage data (such as device interaction logs or wearable sensor data) would provide a more complete picture of the adoption process.

Fifth, K-means clustering relies on several assumptions, most notably that clusters are roughly spherical and comparably sized in Euclidean space, that variables are continuous and on comparable scales, and that Euclidean distances meaningfully represent interindividual similarity. We applied *z* score standardization and PCA-based orthogonalization to partially address these assumptions, and the high stability across 100 random initializations and multiple PCA configurations (94%‐99% agreement) suggests that the identified segments are robust. Nonetheless, the observed clusters were not of equal size (ranging from 16.3% to 45.6%), and some constructs exhibited skewed distributions. Alternative clustering approaches such as Gaussian mixture models or density-based methods (eg, DBSCAN) may be worth examining in future replications to further probe the robustness of the proposed segmentation.

Finally, although the 4-cluster solution was supported by both the elbow method and the silhouette coefficient and demonstrated robust stability across random initializations and PCA configurations, the inherent exploratory nature of cluster analysis means that the identified segments should be treated as empirically derived typologies rather than definitive population parameters. Replication studies using independent samples from different cultural contexts would strengthen the external validity of the proposed segmentation framework.

### Conclusions

This study examined the acceptance patterns of digital health care devices among 349 adults aged 65 years and older in the Seoul metropolitan area using PCA and K-means clustering within the framework of the augmented TAM. We identified 4 different acceptance profiles: core adopters, potential adopters, neutral majority, and rejecters. These profiles demonstrate that technology acceptance among older adults cannot be adequately captured by a simple adopter/nonadopter dichotomy. The observed heterogeneity reveals that, even within a well-established theoretical model such as TAM, older adults exhibit diverse acceptance pathways shaped by different configurations of cognitive readiness, operational confidence, health-related motivation, and attitudinal disposition.

A particularly informative finding is the identification of the potential adopters segment (18.3%), a group that recognizes the value of digital health care devices and holds positive attitudes yet reports the lowest self-efficacy and perceived ease of use among all segments. Within the bounds of a cross-sectional clustering design, this “want-to-but-cannot” configuration points to capability-building—rather than attitude change—as a plausible focus for interventions targeting this segment, since the apparent acceptance barriers appear to lie more in operational capability than in persuasion about benefits. By empirically identifying such actionable segments through the integration of theory-based constructs with data-driven clustering, this study provides a framework that can inform policymakers, technology developers, health care professionals, and industry stakeholders seeking to design differentiated strategies for broader and more inclusive adoption of digital health care technologies in aging societies.

## Supplementary material

10.2196/96557 Checklist 1 STROBE (Strengthening the Reporting of Observational Studies in Epidemiology) checklist for cross-sectional studies.

## Abbreviations

COMP: compatibility
DHA: attitude toward digital health care
HE: health empowerment
HTS: health threat susceptibility
IU: intention to use
PC: price consciousness
PCA: principal component analysis
PEOU: perceived ease of use
PRIV: privacy
PU: perceived usefulness
SE: self-efficacy
STROBE: Strengthening the Reporting of Observational Studies in Epidemiology
TAM: Technology Acceptance Model
WCSS: within-cluster sum of squares
