# Supplementary material for: Segmenting Older Adults by Their Acceptance of Digital Health Care Devices: Cross-Sectional Study Using the Augmented Technology Acceptance Model and K-Means Clustering
Source: JMIR Form Res. 2026 Jun 24;10:e96557. doi: 10.2196/96557 (PMC13293229; doi:10.2196/96557)
Supplement: Checklist 1 [file formative-v10-e96557-s001.docx]

**Checklist 1. STROBE Checklist for Cross-Sectional Studies**

*Manuscript: Segmenting Older Adults by Their Acceptance of Digital Healthcare Devices: Cross-Sectional Study Using the Augmented Technology Acceptance Model and K-Means Clustering*

*JMIR Formative Research, Manuscript #96557*

**STROBE Statement — Checklist of items that should be included in reports of cross-sectional studies**

Give information separately for exposed and unexposed groups (not applicable to this single-sample study)

| **Section/Topic** | **Item #** | **Recommendation** | **Reported on page/section** |
| --- | --- | --- | --- |
| **Title and abstract** |  |  |  |
| **Title** | **1a** | Indicate the study's design with a commonly used term in the title or the abstract | Title; Abstract (Methods) — "Cross-Sectional Study" in title; "A cross-sectional survey was conducted..." in Abstract |
| **Abstract** | **1b** | Provide in the abstract an informative and balanced summary of what was done and what was found | Abstract (Background, Objective, Methods, Results, Conclusions sections included) |
| **Introduction** |  |  |  |
| **Background/rationale** | **2** | Explain the scientific background and rationale for the investigation being reported | Introduction |
| **Objectives** | **3** | State specific objectives, including any prespecified hypotheses | Introduction, research questions (RQ1, RQ2, RQ3) at end of Introduction |
| **Methods** |  |  |  |
| **Study design** | **4** | Present key elements of study design early in the paper | Methods (Study Design) — "This study used a cross-sectional survey design..." |
| **Setting** | **5** | Describe the setting, locations, and relevant dates, including periods of recruitment, exposure, follow-up, and data collection | Methods (Recruitment and Participants) — "Data were collected between March 31, 2025, and April 15, 2025... 6 older adult welfare centers and community facilities located in the Seoul metropolitan area of South Korea" |
| **Participants** | **6** | Give the eligibility criteria, and the sources and methods of selection of participants | Methods (Recruitment and Participants) — Adults aged 65 years and older; exclusion: cognitive impairment; purposive convenience sampling through institutional partnerships |
| **Variables** | **7** | Clearly define all outcomes, exposures, predictors, potential confounders, and effect modifiers. Give diagnostic criteria, if applicable | Methods (Measures); Table 1 — Ten constructs defined with item wording and sources |
| **Data sources/ measurement** | **8*** | For each variable of interest, give sources of data and details of methods of assessment (measurement). Describe comparability of assessment methods if there is more than one group | Methods (Measures); Table 1 (construct sources); Table 3 (descriptive statistics & reliability α) |
| **Bias** | **9** | Describe any efforts to address potential sources of bias | Methods (Recruitment and Participants) — trained undergraduate research assistants; exclusion of cognitive impairment; uniform administration protocol; Discussion (Limitations and Directions for Future Research) — convenience sampling bias, gender imbalance, and generalizability discussed |
| **Study size** | **10** | Explain how the study size was arrived at | Methods (Recruitment and Participants) & Results (Principal Component Analysis) — N = 349 after applying exclusion criteria from initial recruitment pool; adequate for PCA/K-means clustering per convention |
| **Quantitative variables** | **11** | Explain how quantitative variables were handled in the analyses. If applicable, describe which groupings were chosen and why | Methods (Measures) — 5-point Likert scale items, averaged by construct; Methods (Data Analysis Procedures) — Z-score standardization before PCA |
| **Statistical methods** | **12a** | Describe all statistical methods, including those used to control for confounding | Methods (Data Analysis Procedures) — PCA with parallel analysis and Kaiser criterion; K-means clustering; elbow method and silhouette coefficient |
| **Statistical methods** | **12b** | Describe any methods used to examine subgroups and interactions | Methods (Data Analysis Procedures) — K-means clustering used to empirically identify subgroups (not predefined); Results (Cluster Profiles) compares cluster-level means across ten constructs |
| **Statistical methods** | **12c** | Explain how missing data were addressed | Methods (Recruitment and Participants) — Cases with full construct-level missingness or severe overall missingness (more than three missing items across all constructs) were excluded; for the retained sample (N = 349), partial item-level missingness (one or two items within a multi-item construct) was handled via available-item mean within each construct (Schafer & Graham, 2002) [ref. 34] |
| **Statistical methods** | **12d** | If applicable, describe analytical methods taking account of sampling strategy | Not applicable — convenience sampling with no post-hoc weighting applied; limitation acknowledged in Discussion (Limitations and Directions for Future Research) |
| **Statistical methods** | **12e** | Describe any sensitivity analyses | Results (Robustness Checks) — (i) 100-seed K-means stability analysis (Silhouette SD = 0.001); (ii) PCA dimensionality sensitivity analysis (94–99% cluster agreement across 2–5 components); (iii) demographic distribution check verifying that gender, age, and education distributions across the four clusters were broadly consistent with the overall sample composition |
| **Results** |  |  |  |
| **Participants** | **13a** | Report numbers of individuals at each stage of study—eg numbers potentially eligible, examined for eligibility, confirmed eligible, included in the study, completing follow-up, and analysed | Results (Principal Component Analysis) — Final analytic sample N = 349; Methods (Recruitment and Participants) describes recruitment from six welfare centers |
| **Participants** | **13b** | Give reasons for non-participation at each stage | Methods (Recruitment and Participants) — Pre-screening exclusion of older adults with cognitive impairment; post-collection exclusions for failing the age criterion (n = 5), construct-level full missingness (n = 5), and severe overall missingness with more than three missing items (n = 2) |
| **Participants** | **13c** | Consider use of a flow diagram | Not provided (sample construction reported narratively) |
| **Descriptive data** | **14a** | Give characteristics of study participants (eg demographic, clinical, social) and information on exposures and potential confounders | Results (Principal Component Analysis); Table 2 Demographic Characteristics (gender, age, education) |
| **Descriptive data** | **14b** | Indicate number of participants with missing data for each variable of interest | Methods (Recruitment and Participants) & Results (Principal Component Analysis) — Within the retained sample (N = 349), partial item-level missingness was minimal and was handled via available-item mean within each multi-item construct, yielding complete construct-level scores for analysis |
| **Outcome data** | **15*** | Report numbers of outcome events or summary measures | Table 3 (descriptive statistics & Cronbach's α); Table 7 (cluster profiles on raw scale); Figure 5 (cluster profiles on standardized scale) |
| **Main results** | **16a** | Give unadjusted estimates and, if applicable, confounder-adjusted estimates and their precision (eg, 95% confidence interval). Make clear which confounders were adjusted for and why they were included | Not applicable to the primary analysis (unsupervised clustering); Results (Determination of the Optimal Number of Clusters)–4.3 report PCA loadings, cluster membership, and standardized/raw means by cluster |
| **Main results** | **16b** | Report category boundaries when continuous variables were categorized | Methods (Data Analysis Procedures) — Cluster boundaries were empirically derived, not pre-specified; K = 4 selected based on elbow and silhouette criteria (Results (Determination of the Optimal Number of Clusters), Table 6) |
| **Main results** | **16c** | If relevant, consider translating estimates of relative risk into absolute risk for a meaningful time period | Not applicable (no risk estimates; cross-sectional clustering study) |
| **Other analyses** | **17** | Report other analyses done—eg analyses of subgroups and interactions, and sensitivity analyses | Results (Robustness Checks) — (i) 100-seed K-means stability; (ii) PCA dimensionality sensitivity (2–5 components); (iii) demographic distribution check across the four clusters (gender, age, education) |
| **Discussion** |  |  |  |
| **Key results** | **18** | Summarise key results with reference to study objectives | Discussion (Summary and Interpretation of Findings) |
| **Limitations** | **19** | Discuss limitations of the study, taking into account sources of potential bias or imprecision. Discuss both direction and magnitude of any potential bias | Discussion (Limitations and Directions for Future Research) — Convenience sampling & Seoul metropolitan context; gender imbalance (79.4% female); cognitive-impairment exclusion; behavioral intention ≠ actual usage; K-means assumptions (sphericity, equal size) explicitly discussed |
| **Interpretation** | **20** | Give a cautious overall interpretation of results considering objectives, limitations, multiplicity of analyses, results from similar studies, and other relevant evidence | Discussion (Cluster Characteristics in Relation to Theory)–5.3 (cluster interpretations); Discussion (The Role of Health Threat Susceptibility) Role of HTS (explicitly cautious); Discussion (Academic Implications) (including clustering-vs-regression comparison) |
| **Generalisability** | **21** | Discuss the generalisability (external validity) of the study results | Discussion (Limitations and Directions for Future Research), first bullet — Explicit caveats about generalization to male older adults, rural residents, and non-Korean cultural contexts |
| **Other information** |  |  |  |
| **Funding** | **22** | Give the source of funding and the role of the funders for the present study and, if applicable, for the original study on which the present article is based | Funding section — Ministry of Education of the Republic of Korea and National Research Foundation of Korea (NRF-2024S1A5C3A02043877) |

*Reference: von Elm E, Altman DG, Egger M, Pocock SJ, Gøtzsche PC, Vandenbroucke JP; STROBE Initiative. The Strengthening the Reporting of Observational Studies in Epidemiology (STROBE) statement: guidelines for reporting observational studies. Lancet. 2007;370(9596):1453-1457. doi:10.1016/S0140-6736(07)61602-X*

*Checklist is available at: https://www.strobe-statement.org/checklists/*
